# Habenula GPR139 is associated with fear learning in the zebrafish

**DOI:** 10.1038/s41598-021-85002-1

**Published:** 2021-03-10

**Authors:** Nisa Roy, Satoshi Ogawa, Roshan Maniam, Ishwar Parhar

**Affiliations:** Brain Research Institute, Jeffrey Cheah School of Medicine and Health Sciences, Monash University Malaysia, 47500 Bandar Sunway, Selangor Malaysia

**Keywords:** Emotion, Learning and memory, Animal behaviour, Ichthyology

## Abstract

G-protein coupled receptor 139 (GPR139) is an evolutionarily conserved orphan receptor, predominantly expressing in the habenula of vertebrate species. The habenula has recently been implicated in aversive response and its associated learning. Here, we tested the hypothesis that GPR139 signalling in the habenula may play a role in fear learning in the zebrafish. We examined the effect of intraperitoneal injections of a human GPR139-selective agonist (JNJ-63533054) on alarm substance-induced fear learning using conditioned place avoidance paradigm, where an aversive stimulus is paired with one compartment, while its absence is associated with the other compartment of the apparatus. The results indicate that fish treated with 1 µg/g body weight of GPR139 agonist displayed no difference in locomotor activity and alarm substance-induced fear response. However, avoidance to fear-conditioned compartment was diminished, which suggests that the agonist blocks the consolidation of contextual fear memory. On the other hand, fish treated with 0.1 µg/g body weight of GPR139 agonist spent a significantly longer time in the unconditioned neutral compartment as compared to the conditioned (punished and unpunished) compartments. These results suggest that activation of GPR139 signalling in the habenula may be involved in fear learning and the decision-making process in the zebrafish.

## Introduction

GPR139 is a brain-rich orphan G-protein coupled receptor, and its structure is conserved across various mammalian and non-mammalian vertebrates and in invertebrate species. The human GPR139 protein has 96%, 92%, 70% and 89% identical homology to the mouse, chicken, zebrafish and *C. elegans* orthologs, respectively^1–3^. In mammals, GPR139 is most predominantly expressed in the habenula^1^, but it is also expressed in other regions such as the lateral septal nucleus, basal ganglia, hypothalamus and locus coeruleus^1,4–6^. Similarly, recent single-cell transcriptome analysis in the zebrafish revealed discrete expression of *gpr139* gene in the habenula^7^. Although GPR139 is still an orphan receptor, the potential role of GPR139 signalling has been elucidated using synthetic selective agonists and antagonists for GPR139^8,9^. Male rats administered with a GPR139 agonist (JNJ‐63533054) exhibit hyperlocomotion and anhedonic-like behavioural change^1,10^. A recent study using *Gpr139* gene-knockout mice has shown the involvement of GPR139 in the modulation of morphine-induced analgesia, reward, and withdrawal^6^. However, because of the widespread distribution of GPR139 in the brain of mammals, the use of selective GPR139 agonist or antagonist indiscriminately targets several GPR139 expression sites in the brain. Hence, the specific physiological role of GPR139 signalling in each brain region, particularly in the habenula remains obscure at present.

The habenula is an evolutionarily conserved brain structure in the epithalamus of vertebrates^11^. In mammals, the habenula comprises two subnuclei, the medial (MHb) and lateral habenula (LHb) with specific morphological, biochemical and molecular characteristics^12,13^. In fish and amphibians, the habenula consists of the dorsal (dHb) and ventral habenula (vHb), which corresponds to mammalian MHb and LHb, respectively^14,15^. Habenula plays crucial roles in processing emotional and aversive responses^16,17^, decision-making^16^, and it has also been implicated in the pathophysiology of neuropsychiatric disorders^18,19^. Recently, the habenula has also been shown to be involved in aversive learning and memories^20^. In rats, lesions of the LHb enhances performance in avoidance learning^21^. On the contrary, selective inactivation of LHb disrupts temporal stability of fear memory^22^. Similarly, mice with selective ablation of MHb exhibit deficits in aversive learning and spatial memory^23^. In fish, the habenula has also been implicated in aversive responses and its associated learning^24^. In zebrafish, the dHb is involved in the experience-dependent modification of aversive responses, including fear responses and social conflict resolution^24–26^. We have previously shown the expression of a neuropeptide, kisspeptin 1 (Kiss1) in the vHb and its modulatory role in the odorant cue (alarm substance) induced fear-like responses in the zebrafish^27,28^. A more recent study has shown that genetic ablation of *kiss1* impairs aversive learning in larval zebrafish^29^. Furthermore, we have recently demonstrated the possible involvement of habenula Kiss1 in opioid-induced fear learning impairment^30^. Similarly, optogenetic stimulation of vHb neurons alone can evoke conditioned place avoidance in zebrafish^31^. These results affirm the role of the vHb pathway in aversive learning in zebrafish. Hence, we hypothesise that GPR139 in the vHb may play a role in modulating aversive learning in zebrafish. To challenge this hypothesis, in the present study, using a human GPR139-selective synthetic agonist (JNJ-63533054)^1,9^, we examined the possible role of GPR139 signalling in fear associated memory consolidation using the conditioned place avoidance paradigm^32^.

## Results

### Expression of *gpr139*mRNA in the brain

**Figure 1:**
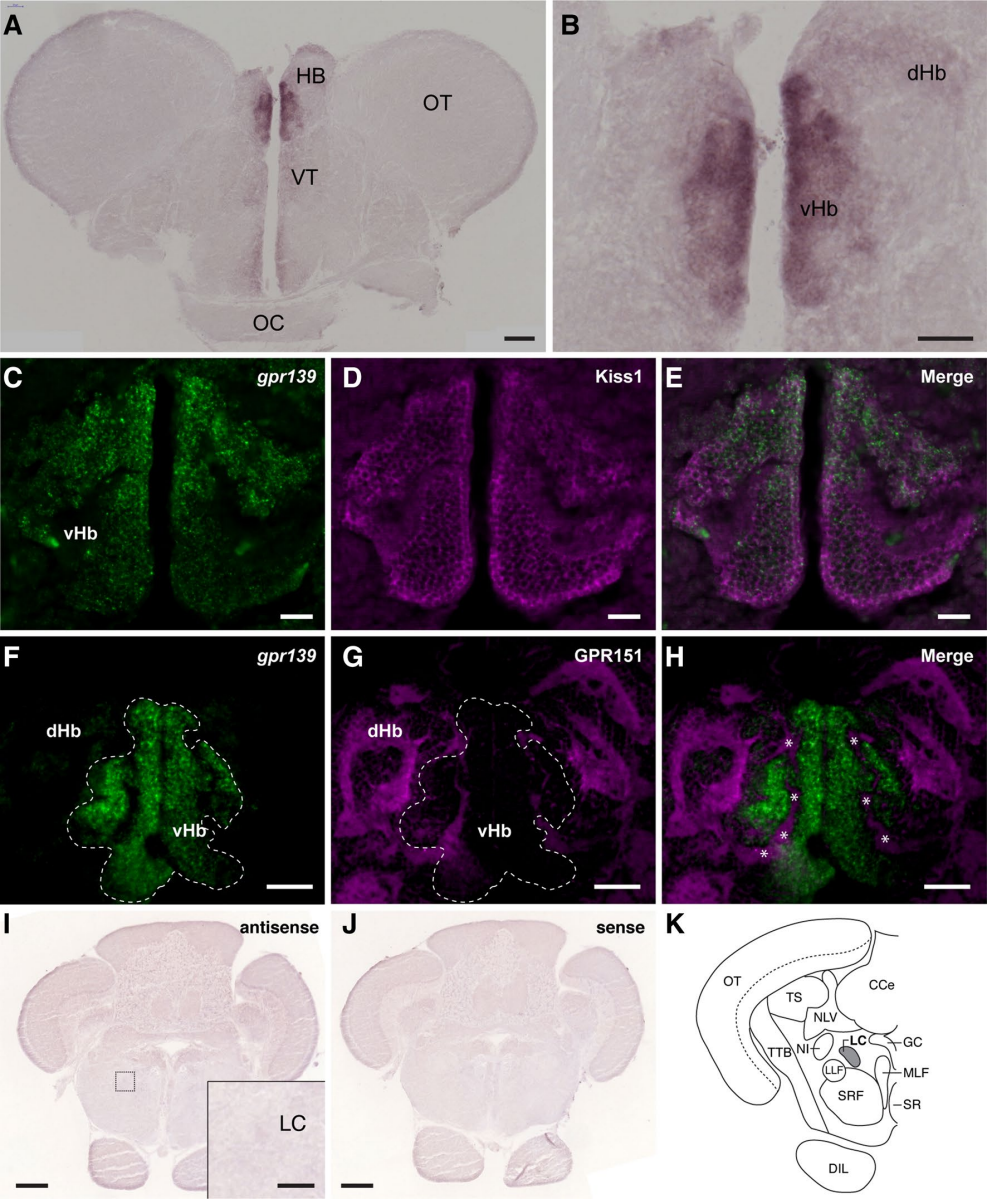
Localisation of *gpr139* mRNA expression in the brain of zebrafish. In situ hybridisation shows expression of *gpr139* mRNA in the ventral habenula (vHb) (**A**, **B**). Double labelling of *gpr139* mRNA (**C**, **F**) with Kiss1 (**D**) or GPR151(**G**) immunofluorescence further confirmed specific expression of *gpr139* mRNA in the vHb (**E**) but not in the dorsal habenula (dHb) (**H**). In the vHb, GPR151-immunoreactive neuropil structure is also seen, which are derived from the dHb (**H**, *asterisks*). In the hindbrain region, no expression of *gpr139* mRNA was detected in the locus coeruleus (**I**–**K**). The dotted box indicates the location for the inset in the panel I. *HB* habenula; *VT* ventral thalamus; *OT* optic tectum; *OC* optic chiasma; *dHb* dorsal habenula; *vHb* ventral habenula; *LC* locus coeruleus; *TS* torus semicircularis; *CCe* cerebellar corpus; *NLV* nucleus lateralis valvulae; *GC* central gray; *MLF* nucleus of the medial longitudinal fascicle; *NI* nucleus isthmi; *LLF* lateral longitudinal fascicle; *SRF* superior reticular formation; *SR* superior raphe nucleus; *TTB* tecto-bulbar tract; *DIL*, diffuse nucleus of inferior lobe. Scale bars: (**A**, **F**–**H**), 100 µm; (**B**) inset of (**I**), 50 µm; (**C**–**E**), 20 µm; (**I**, **J**), 200 µm.

In situ hybridisation showed discrete expression of *gpr139* mRNA in the vHb (Fig. 1A,B, and Supplementary Figs. S1 and S2). Double-labelling showed co-localisation of *gpr139* mRNA and Kiss1 immunoreactivity in the vHb. However, *gpr139* expression appeared to be concentrated in the dorsal part of the vHb (dorso-vHb) (Fig. 1C–E). In addition, there was no co-expression of *gpr139* mRNA and immunoreactivities to GPR151, a selective marker for the dHb, confirming specific expression of *gpr139* mRNA in the vHb (Fig. 1F–H). In the hindbrain, there was no expression of *gpr139* mRNA in the locus coeruleus (Fig. 1I–K, and Supplementary Figs. S1 and S2).

### Response of zebrafish GPR139 to JNJ-63533054

**Figure 2:**
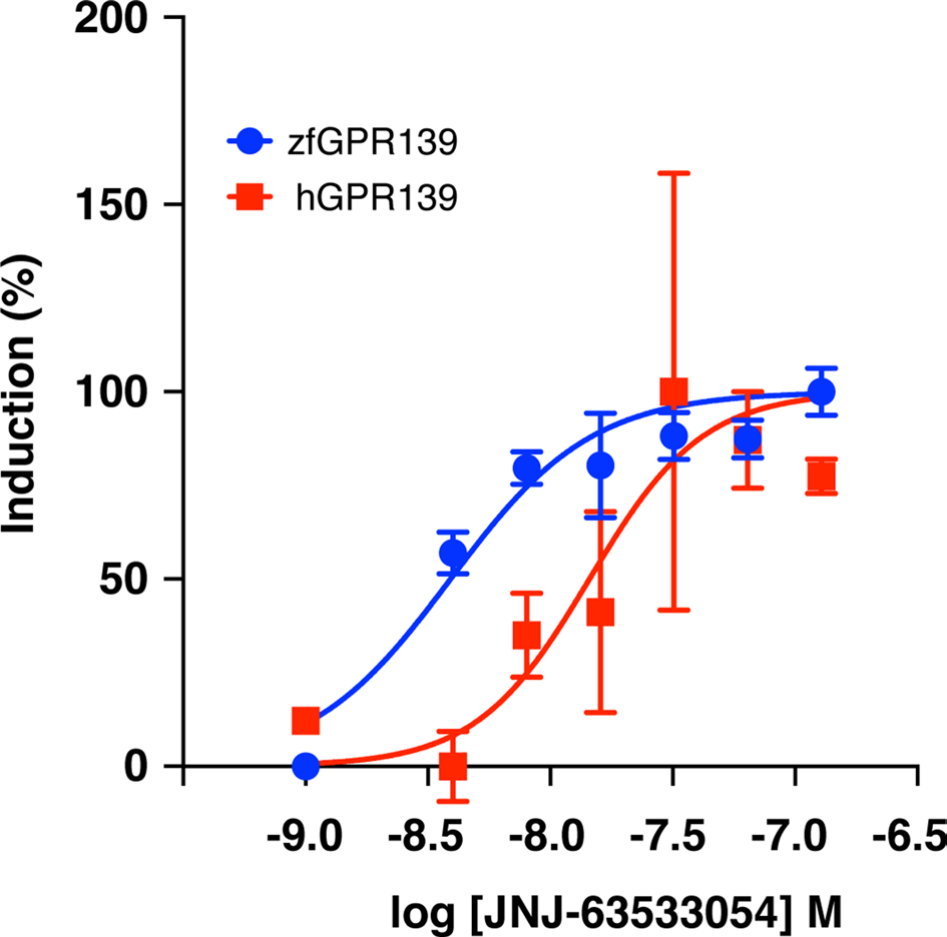
Dose–response curves for luciferase induction by GPR139 agonist JNJ-63533054 upon zebrafish GPR139 in HEK293-T cells. Graphs showing concentration-dependent induction firefly luciferase activity divided by Renilla luciferase activity (%) in the HEK 293 T cells expressing zebrafish GPR139 (blue) and human GPR139 (red) by JNJ63533054 (4 ~ 128 nM). The EC50 values (3.91 nM and 14.45 nM for zebrafish GPR139 and human GPR139, respectively) were calculated by nonlinear regression analysis of the dose–response curves generated using the Prism 9 program. All data points are representative of three independent experiments performed in duplicate.

The binding affinity of a selective agonist for human GPR139 (JNJ-63533054) on zebrafish GPR139 was analysed by dual-luciferase reporter assay. JNJ-63533054 induced luciferase activity against zebrafish GPR139 in a concentration-dependent manner (Fig. 2). For zebrafish GPR139, the maximal induction of 3.3-fold of vehicle control was achieved at a concentration of 128 nM. From the dose–response curve, the half effective maximal concentration (EC50) values of JNJ-63533054 to zebrafish GPR139 was 3.91 nM. Analysis of responses of three replicates shows that JNJ-63533054 induced luciferase activity with an inter-assay coefficient of variability (CV) of 4.74% and intra-assay CV of 8.40%. For human GPR139, the maximal induction of 1.5-fold of vehicle control was achieved at the concentration of 64 nM. From the dose–response curve, the EC50 values of JNJ-63533054 to human GPR139 was 14.45 nM, which is relatively similar to EC50s of JNJ-63533054 that were previously reported (16 ~ 17 nM)^1,9^ (Fig. 2).

### Effect of GPR139 agonist on locomotion

**Figure 3:**
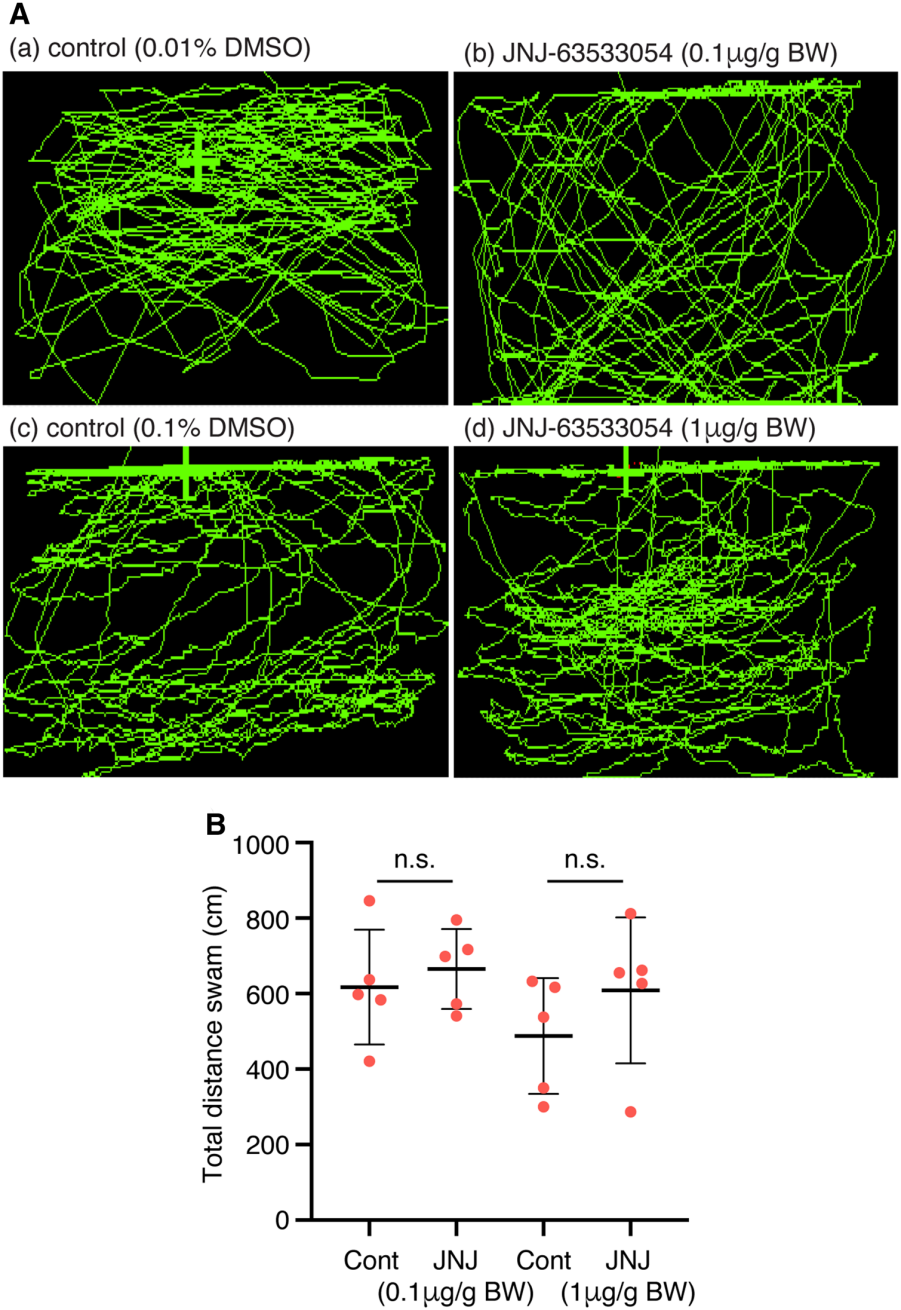
Effect of GPR139 agonist (JNJ63533054) on locomotion. (**A**) Side-view video tracking of swimming behaviour in the observation tank after administration of vehicle controls (**a**, 0.01% DMSO; **c**, 0.1% DMSO) and GPR139 agonist (**b**, 0.1 µg/g; **d**, 1 µg/g body weight, BW). (**B**) Effect of GPR139 agonist on locomotor activity was assessed by analysing total distance swam in the tank, but there was no significant (n.s.) effect of GPR139 agonist (0.1 µg/g, *P* = 0.5784; 1 µg/g, *P* = 0.3053) on locomotor activity.

After administrating the GPR139 agonist at two different doses (0.1 µg/g and 1 µg/g body weight), the fish were transferred to an open-tank once they recovered from anaesthesia, and the locomotor activity of the experimental fish was measured for 6-min (Fig. 3A). However, there was no significant difference in the locomotor activity between fish treated with controls and GPR139 agonist [0.1 µg/ body weight (BW), *P* = 0.5784, Cohen’s *d* = 0.3655; 1 µg/BW, *P* = 0.3053, Cohen’s *d* = 0.6923; Fig. 3B].

### Effect of GPR139 agonist on alarm substance (AS)-induced fear response

**Figure 4:**
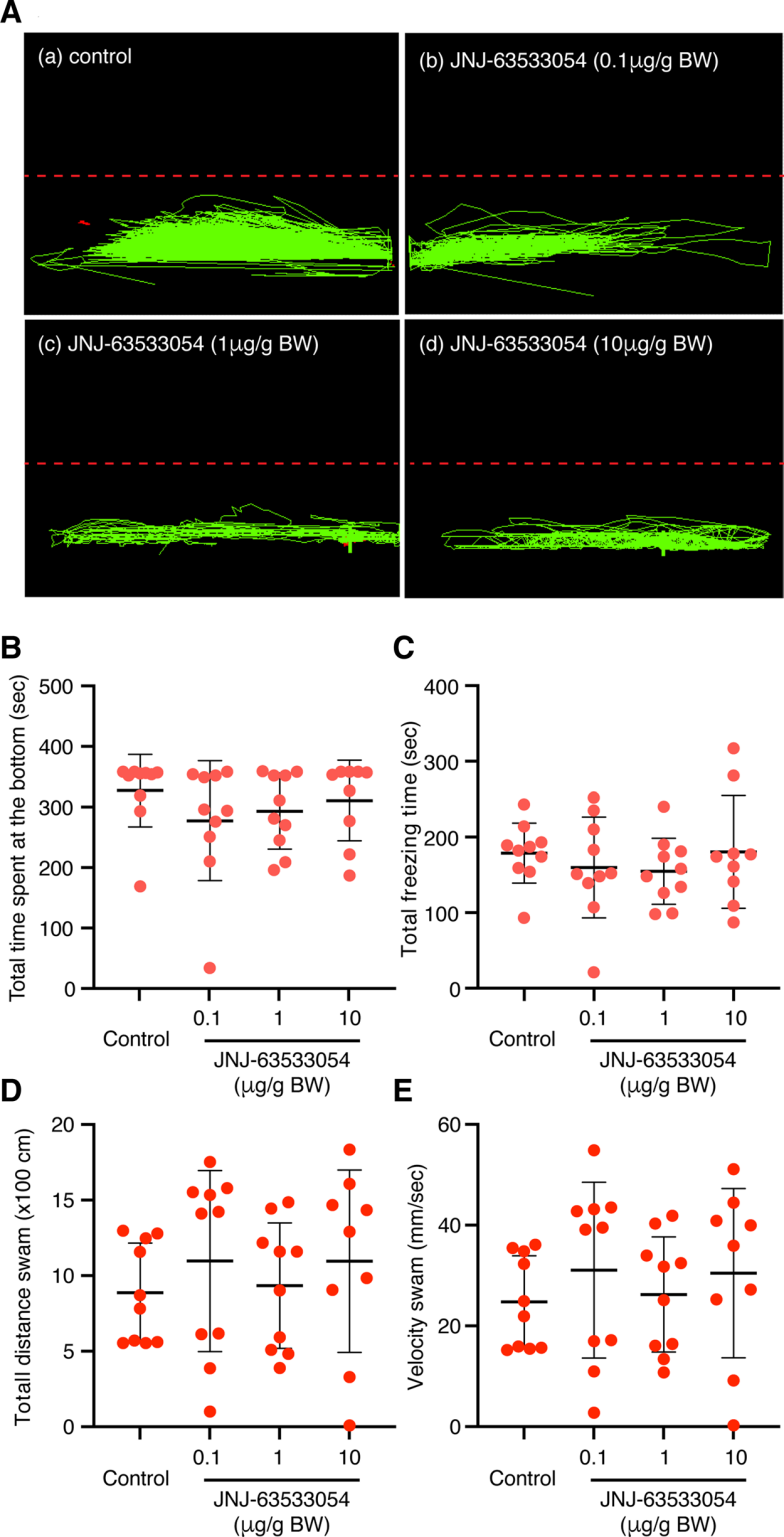
Effect of GPR139 agonist (JNJ63533054) on alarm substance-induced fear response. (**A**) Representative side-view video tracking of swimming behaviour at 30 min after administration of GPR139 agonist (**b**, 0.1 µg/g; **c**, 1 µg/g; **d**, 10 µg/g body weight, BW) and subsequent alarm substance (AS) administration. However, there were no significant differences in AS-induced fear responses including the total time spent (*P* = 0.4789, **B**), total freezing time (*P* = 0.6826, **C**), total distance swam (*P* = 0.7077, **D**), and swimming velocity (*P* = 0.6975, **E**) at the bottom of a tank among GPR139 agonist treated and control groups.

In controls, immediately after alarm substance (AS)-exposure, the fish exhibited typical AS-induced fear responses including an increase in the number of erratic movements, duration of freezing, and time spent in the lower compartment compared to the upper compartment (Fig. 4A). There were no differences in AS-induced fear responses including the time spent in the lower compartment [F_(3, 35)_ = 0.8445, *P* = 0.4789, gεs = 0.07, 95% CI (0.00, 0.22), Fig. 4B], duration of freezing [F_(3, 35)_ = 0.5031, *P* = 0.6826, gεs = 0.04, 95% CI (0.00, 0.16), Fig. 4C], total distance swam [F_(3, 35)_ = 0.4662, *P* = 0.7077, gεs = 0.04, 95% CI (0.00, 0.16), Fig. 4D] and swimming velocity [F_(3, 35)_ = 0.4811, *P* = 0.6975, gεs = 0.04, 95% CI (0.00, 0.16), Fig. 4E] at the bottom of the tank between fish treated with three doses (0.1 µg/g, 1 µg/g and 10 µg/g body weight) of GPR139 agonist (JNJ-63533054) and control (distilled water, DW). There was no dose-dependent difference among fish treated with three doses of GPR139 agonist (Fig. 4B–E).

### Effect of GPR139 agonist on conditioned place avoidance

**Figure 5:**
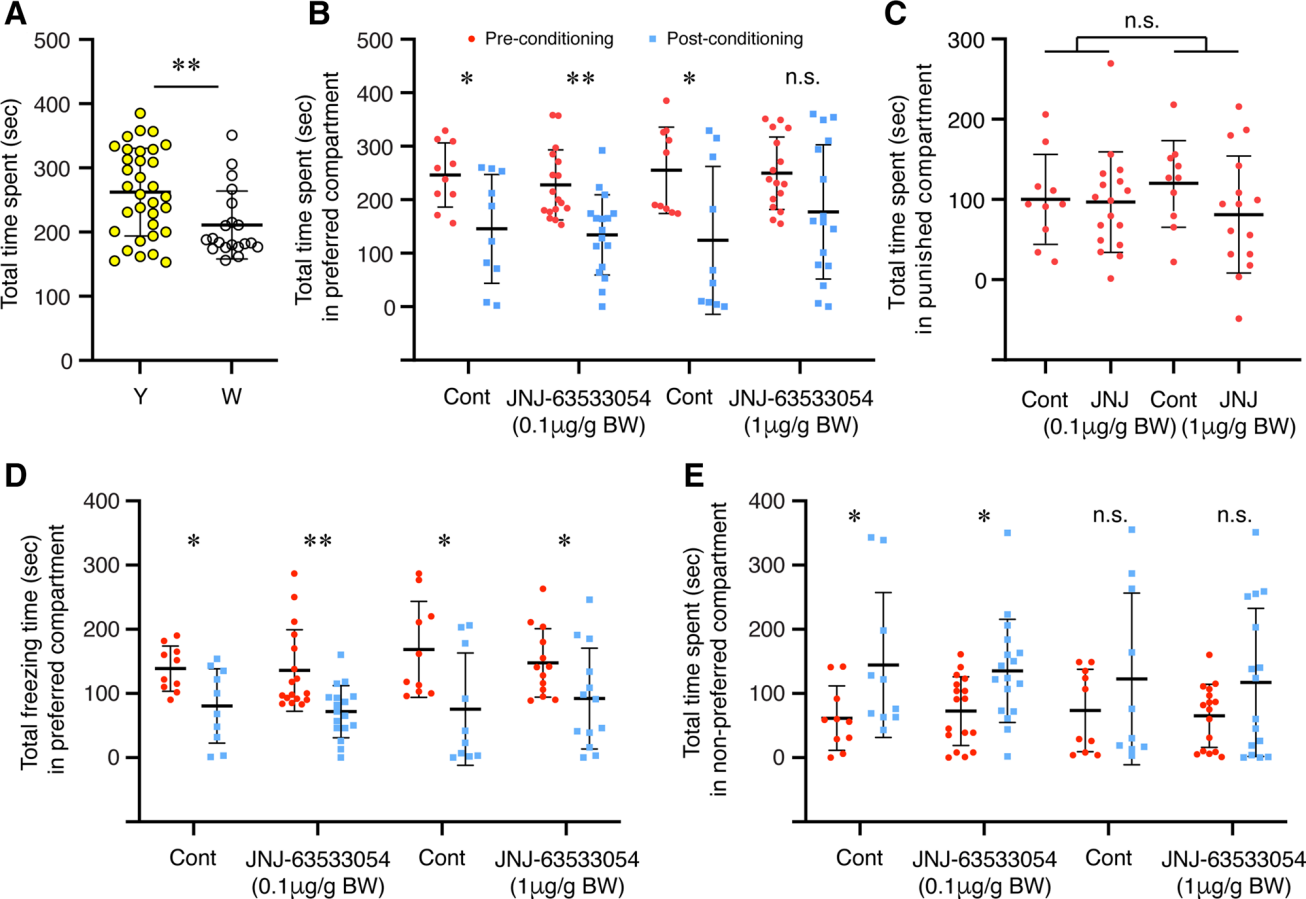
Effect of GPR139 agonist on fear memory recall and avoidance. (**A**) During pre-conditioning, fish were given a choice for their preferred colour, either yellow (Y) or white (W) coloured compartment. In total, a significantly higher number of fish preferred yellow coloured compartment during the pre-conditioning (*P* = 0.006). After conditioning to alarm substance (AS)-induced fear-like responses, the fish were administered with GPR139 agonist (JNJ-63533054, either 0.1 or 1 µg/g body weight, BW), and the following day (Day-3), their preference was assessed based on their total time spent in either AS-paired (originally preferred) or water-paired (originally non-preferred) compartment. (**B**) During the post-conditioning, the time spent in AS-paired compartment (blue dots) was significantly reduced in both controls (*P* = 0.0147 and *P* = 0.0185) and fish treated with 0.1 µg/g BW of GPR139 agonist (*P* = 0.0005) as compared to pre-conditioning period (red dots), indicating successful development of conditioned place avoidance. On the other hand, in fish treated with the higher dose (1 µg/g BW) of GPR139 agonist, there was no difference in the time spent in the preferred compartment between pre-conditioning and post-conditioning phase (*P* = 0.0519). (**C**) There was no dose-dependency in place avoidance (*P* = 0.8403). (**D**) During the post-conditioning period, the total time frozen in the AS-paired compartment was significantly reduced in all groups [controls (0.2% DMSO, *P* = 0.0143; 2% DMSO, *P* = 0.0197), 0.1 µg/g BW GPR139 agonist (*P* = 0.0013), 1 µg/g BW GPR139 agonist (*P* = 0.0459)]. (**E**) On the other hand, the time spent in the water-paired unpunished compartment (originally non-preferred compartment) was increased during the post-conditioned phase in control (0.2% DMSO, *P* = 0.0483, Cohen’s *d* = 0.9472) and the fish treated with 0.1 µg/g BW GPR139 agonist (*P* = 0.0118, Cohen’s *d* = 0.9155). However, in control (2% DMSO) and the fish treated with 1 µg/g BW GPR139 agonist, there was no significant change in the time spent in the water-paired unpunished compartment after the fear conditioning (control, *P* = 0.3086, Cohen’s *d* = 0.4686; 1 µg/g BW GPR139 agonist, *P* = 0.1072, Cohen’s *d* = 0.5877). *, *P* < 0.05; **, *P* < 0.01; n.s., no significant difference.

To investigate the possible role of habenula GPR139 signalling in fear learning, AS-conditioned place avoidance was examined in fish treated with GPR139 agonist. During the pre-conditioning period, the fish were given choices, either while or yellow compartment, and their total time spent over 50% was considered their basal preference. The fish tested spent a significantly longer period in the yellow compartment than the white compartment (*P* = 0.0060, Cohen’s *d* = 0.8381, Fig. 5A). Control fish [administered with distilled water (DW) containing 0.2% or 2% of dimethyl sulfoxide (DMSO)] exposed to AS during the conditioned period significantly reduced their time spent in the preferred compartment during the post-conditioning period (0.2% DMSO: *P* = 0.0147, Cohen’s *d* = 1.2058; 2.0% DMSO: *P* = 0.0185, Cohen’s *d* = 1.1583), indicating that AS-exposure experience (on day-2) successfully induced active avoidance to the AS-paired compartment (Fig. 5B). The fish treated with 0.1 µg/g BW of GPR139 agonist exhibited clear avoidance to the AS-paired compartment during the post-conditioning phase (*P* = 0.0005, Cohen’s *d* = 1.3265, Fig. 5B). On the other hand, the time spent in the water-paired unpunished compartment (originally non-preferred compartment) was increased during the post-conditioned phase in control (0.2% DMSO, *P* = 0.0483, Cohen’s *d* = 0.9472) and the fish treated with 0.1 µg/g BW GPR139 agonist (*P* = 0.0118, Cohen’s *d* = 0.9155) (Fig. 5E). There was no concentration-dependent effect of GPR139 agonist on active avoidance to AS-paired compartment during the post-conditioning phase [F_(1, 46)_ = 0.0411, *P* = 0.8403, gεs = 0.00, 95% CI (0.00, 0.08)] (Fig. 5C). During the post-conditioning period, the total time frozen in the AS-paired compartment was significantly reduced in all groups [controls (0.2% DMSO, *P* = 0.0143, Cohen’s *d* = 1.2123; 2% DMSO, *P* = 0.0197, Cohen’s *d* = 1.1441), 0.1 µg/g BW GPR139 agonist (*P* = 0.0013, Cohen’s *d* = 1.2055); 1 µg/g BW GPR139 agonist (*P* = 0.0459, Cohen’s *d* = 0.8258)], which is because of the conditioned place avoidance (Fig. 5D).

**Figure 6:**
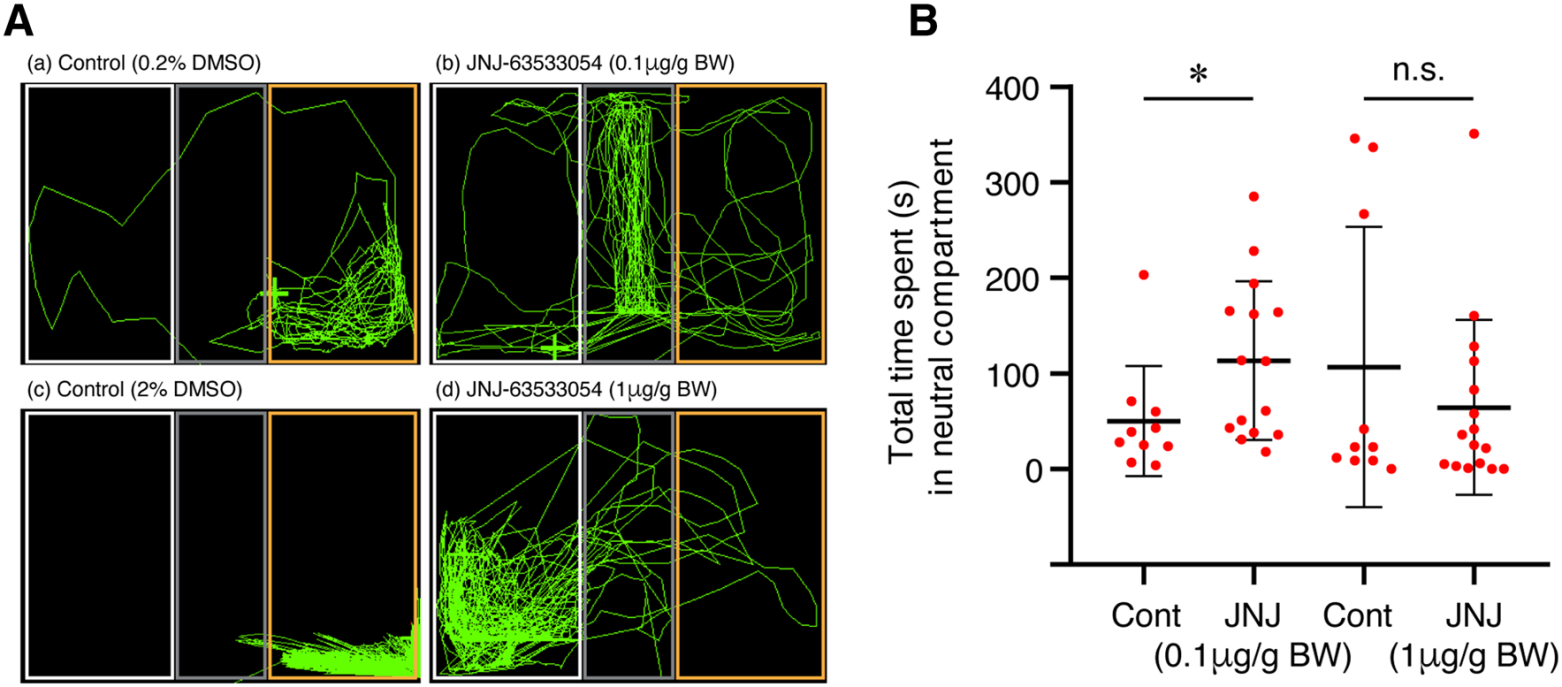
Effect of GPR139 agonist on preference to the neutral compartment during fear memory recall. (**A**) Representative top-view video tracking of swimming behaviour in controls (**a** and **c**, 0.2% and 2% DMSO, respectively) and the fish treated with GPR139 agonist (**b** and **d**, 0.1 and 1 µg/g BW of JNJ-63533054, respectively). (**B**) Total time spent in the neutral context (grey compartment) during post-conditioning in controls and GPR139 agonist-treated fish. The fish treated with 0.1 µg/g BW of GPR139 agonist spent a significantly (*P* = 0.0477) longer time in the neutral compartment as compared to control. *, *P* < 0.05. n.s., no significant difference.

In the fish treated with 1 µg/g BW of GPR139 agonist, there was no significant difference in total time spent in the AS-paired compartment during the post-conditioning phase (*P* = 0.0519, Cohen’s *d* = 0.7163, Fig. 5C). In addition, there was no significant difference in the time spent in the water-paired unpunished compartment in control (2% DMSO, *P* = 0.3086, Cohen’s *d* = 0.4686) and the fish treated with 1 µg/g BW of GPR139 agonist (*P* = 0.1072, Cohen’s *d* = 0.5877) between pre- and post-conditioning phase (Fig. 5E). These results indicate that the conditioned place avoidance was impaired in the fish treated with 0.1 µ/g BW of GPR139 agonist. In the fish treated with 0.1 µ/g BW of GPR139 agonist, their time spent in the neutral (middle in grey colour) compartment during the post-conditioning phase was significantly longer (*P* = 0.0477, Cohen’s *d* = 0.8851) as compared to controls and 1 µ/g BW of GPR139 agonist-treated group (*P* = 0.3728, Cohen’s *d* = 0.3454) (Fig. 6A,B).

## Discussion

Despite the conserved gene structure of GPR139 across vertebrates, its expression pattern has topographic differences in the habenula. In our study in the zebrafish, *gpr139* gene is discretely expressed in the vHb, the homolog of the mammalian LHb^15^. In contrast, in rodents, GPR139 is expressed mainly in the MHb and lesser in the LHb^1,4^. In addition to the differential expression of GPR139, expression patterns of other habenula-specific markers are also different between rodents and zebrafish. For instance, GPR151 (an orphan GPCR) and Brn3a (POU domain, class 4, transcription factor 1), are expressed in the LHb and MHb in rodents^33,34^. On the other hand, in the zebrafish, *gpr151* ortholog and *brn3a* are only expressed in the dHb^14,33^. More specifically, in our study, the expression of *gpr139* mRNA is seen in the dorsal part of the vHb, suggesting that the vHb in the zebrafish is a heterogeneous subpopulation of cells. Topographic connections reveal that the vHb selectively sends an efferent pathway to the median raphe via the fasciculus retroflexus^15^. The localisation of *gpr139* mRNA-expressing neurons in the dorso-vHb suggests this subnucleus of the vHb might project to a specific subset of neurons in the median raphe^35^. These studies indicate that the cytoarchitecture of the habenula in the zebrafish is either simpler as compared to that in mammals^12^ or more complex structure of several subnuclei, which remain to be identified. In rodents, GPR139 is also expressed in the locus coeruleus (LC)^6^. However, based on the morphological characterisation of the LC in the zebrafish^36^, we confirmed the absence of *gpr139* mRNA expression in the LC. We also found some antisense-labelled signals outside of the habenula (see Supplementary Figs. S1 and S2). However, those signals appear to be weak compared to the signals in the vHb, which can be considered negligible levels based on comparison to replicate sections that were hybridized with the sense probe (Supplementary Figs. S1 and S2). Thus, the effect of GPR139 agonist on fear learning is mostly modulated by GPR139 in the vHb, and possible involvement of weakly expressed GPR139 outside of the habenula is unlikely. Hence, considering the discrete expression of *gpr139* in the vHb, the zebrafish could be an ideal model to elucidate the specific role of GPR139 signalling within the habenula. However, a recent study in rats has shown expression of *Gpr139* mRNA in several other brain regions including the ventral tegmental area, olfactory tubercle, substantia nigra, and the hippocampus using a sensitive RNA probe (RNAscope)^6^. Thus, the possible role of GPR139 signalling outside of the habenula remains to be further validated.

To examine the possible role of GPR139 in the habenula and its related functions in the zebrafish, we first examined if a commercially available human GPR139 agonist, JNJ-63533054 (also known as compound 7c)^1,9^ exhibits an agonistic response against zebrafish GPR139. The luciferase assay revealed that JNJ-63533054 effectively binds and acts as an agonist to zebrafish GPR139. Further, based on EC50, JNJ-63533054 exhibits approximately 4-folds higher potency in zebrafish GPR139 as compared to human GPR139^1,9^, affirming JNJ-63533054 as a reliable agonist for zebrafish GPR139. In rats, oral administration of JNJ-63533054 suppresses locomotor activity^1^. However, treatment with JNJ-63533054 did not alter locomotor activity in the zebrafish. In rodents, expression of GPR139 in the striatum has been implicated in the control of locomotor activity^2,8^. Although the location of striatum-like structure in the brain of teleosts is unclear, neurochemical and morphological characterisation suggested the dorsal and central part of the ventral telencephalon as the putative teleostean homolog of striatum^37,38^. In addition, in rodents, the MHb, where GPR139 is also highly expressed^1,39^, has also been implicated in the control of locomotion^40,41^. However, in the zebrafish *gpr139* mRNA is expressed in neither the ventral telencephalic regions nor the dHb. These results suggest that GPR139 in the vHb is not involved in locomotor activity in the zebrafish. Further, a recent study showed no impairment in the locomotion and motor coordination in *Gpr139* knockout mice^6^, hence, the effect of GPR139 agonist/antagonist on the locomotion remains to be further validated. A recent study demonstrated the role of GPR139 in opioid sensitivity in the brain of mice^6^, where GPR139 is co-expressed with mu-opioid receptor (MOR) in the MHb and the LC, and it forms heterodimerisation with MOR to inhibit MOR signalling. However, this may not be the case for the zebrafish, at least at the central nervous system level, as MOR (*oprm1*) mRNA is mainly expressed in the dHb but not in the vHb^42^. Nevertheless, we have recently demonstrated the possible involvement of vHb in opioid-induced fear memory impairment in zebrafish^30^. Thus, it is possible that GPR139 signalling could play a role in opioid-sensitivity and its associated memory impairment to some extent. Although the expression of *gpr139* outside of the brain remains unexamined in the zebrafish, the role of GPR139 in opioid sensitivity could be facilitated in the peripheral system as similar to mammals.

We have previously shown the expression of Kiss1 in the vHb and its role in AS-induced fear-like response in the zebrafish^27^. However, in the present study, administration of GPR139 agonist did not affect AS-induced fear-like response. These results indicate that the role of GPR139 signalling is independent of the Kiss1 signalling in the vHb. The habenula has recently been implicated in aversive and spatial memory^22,43^ and decision making^16^. However, the low dose GPR139 agonist treated fish showed apparent avoidance to the fear-conditioned compartment and increased in entry to the unpunished compartment. On the other hand, fish treated with the higher dose (1 µg/g BW) of GPR139 agonist did not show an apparent conditioned avoidance towards the aversively conditioned compartment, and there was no preference change towards the unpunished compartment after the fear experience. These results suggest that the high dose of GPR139 agonist blocks the consolidation but not the acquisition of contextual fear memory. However, in the control group (2% DMSO), there was no increase in the entry to the unpunished compartment during the post-conditioning, despite a significant reduction in the time spent in the punished compartment. These results may imply that a higher concentration of DMSO might have influenced the fear memory retrieval process, which remains to be further validated.

Interestingly, the fish treated with the lower dose (0.1 µg/g BW) of GPR139 agonist exhibited a preference to the neutral (unconditioned, grey-coloured) compartment and avoided the preferred and non-preferred (either yellow or white in colour) fear-conditioned compartments. Some teleost species including the zebrafish have an innate preference for specific colours^44–46^. In the present study, fish exhibited some biasness for yellow-coloured compartment during the pre-conditioned phase. However, treatment with GPR139 agonist did not affect their colour preference or avoidance. Although GPR139 agonist treatment had no unconditional anxiogenic effect, there was no effect of GPR139 agonist on the conditioned place avoidance (decrease in entry to the punished compartment and increased in entry to the unpunished compartment). Alternatively, these individuals may exhibit a risk-avoidance behaviour due to higher sensitivity to AS-induced aversion, which in turn might have resulted in excessive avoidance of high-risk compartments or preference to a safe zone (middle compartment). In mice, MHb ablation induces several cognitive impairments including delay and effort aversion in a decision-making test, deficits in spatial memory and a subtle increase in anxiety levels^23^. Furthermore, in humans, genetic variations in *GPR139* locus have been linked to schizophrenia^47^ and symptoms of inattention in attention deficit hyperactivity disorder^48^. These neurological disorders associated with cognitive impairments are also implicated with decision-making impairment^49^. Thus, it can be speculated that in GPR139 agonist treated fish, their risk-associated decision-making process might have been partially compromised.

Although GPR139 agonist treatment appears to exhibit a dose-dependent effect on fear memory consolidation and retrieval process, the possible mechanism of this phenomenon remains unknown. It has been shown that GPR139 is a dual-specificity receptor capable of activating G proteins of the G_i/o_ and G_q/11_ classes upon application of 10 μM JNJ-63533054 in GPR139-transfected HEK293 cells^50^. However, GPR139 primarily engages the G_q/11_ but not G_i/o_ pathway to activate adenylyl cyclase and inhibit the G protein inward rectifying potassium (GIRK) channel^50^. A recent study showed that unpredictable aversive stress (food-shock) reduces GABA_B_ receptor-GIRK signalling via triggering internalisation of GABA_B_-GIRK, causing an increase in neuronal excitability and depressive-like behaviours in mice^51^. In mice, contingent association of an auditory cue with a punishment progressively causes cue-driven LHb neuronal excitation during avoidance learning^52^. Similarly, vHb neurons have been shown to increase activity in response to the aversive cue in the zebrafish^31^. This induction of neuronal activity is suggested to represent the expectation of an aversive outcome and be used for comparison with a real outcome when the fish is learning how to escape from a dangerous to a safer environment^31^. Hence, activation of GPR139 signalling by JNJ-63533054 might have influenced its downstream signalling such as inhibition of GIRK during the fear memory consolidation or retrieval process. A previous study in zebrafish showed that Kiss1 peptide treatment induces depolarisation of vHb neurons at low concentrations and hyperpolarisation at high concentrations^29^. Hence, the GPR139 agonist may exhibit a similar effect on vHb neurons, where the two different doses applied differentially alter the firing properties of vHb neurons, which might have influenced on aversive expectation value of the contextual conditions.

In summary, we have demonstrated a discrete expression of *gpr139* mRNA in the dorso-vHb in the zebrafish. Administration of a high dose of human GPR139-selective agonist (JNJ‐6353305) had no effect on locomotor activity, and fear response, but fear-conditioned place avoidance was diminished. On the other hand, fish treated with a lower dose of GPR139 agonist exhibited avoidance to the contextual compartments, suggesting the possible involvement of GPR139 signalling in the vHb in fear memory consolidation or memory-based decision-making in the zebrafish.

## Materials and methods

### Animals and housing

Sexually mature (> 6 months old) male, AB wild-type zebrafish (*Danio rerio*) were obtained from the Institute of Molecular and Cell Biology, Singapore. Fish were maintained in groups of 10 fish per 20 L freshwater aquaria (home tank) at 28 ± 0.5 °C with a controlled natural photo regimen (14/10 h, light/dark) at the Brain Research Institute Fish Facility, Monash University Malaysia. The fish were fed with Adult Zebrafish Diet (Zeigler, Gardners, PA, USA) twice daily. All experiments were carried out only after 1 week of fish acclimatisation. The fish were anesthetised by immersion in water containing benzocaine (0.1 g benzocaine/200 mL water; Sigma) before the injections and the dissection of tissues.

### Ethical statement

This study was carried out in strict accordance with the recommendations in the Guidelines to promote the wellbeing of animals used for scientific purposes: The assessment and alleviation of pain and distress in research animals (2008) by the National Health and Medical Research Council of Australia (https://www.nhmrc.gov.au/guidelines-publications/ea18). All experimental protocols and procedures were approved by the Animal Ethics Committee of Monash University Malaysia (Project Approval Number: 2019–18719-34397).

### Expression of *gpr139* mRNA in the zebrafish brain

Expression of *gpr139* mRNA in the brain was examined by in situ hybridisation following the procedure described previously^53^. The brains of male zebrafish (n = 3) were fixed in buffered 4% paraformaldehyde for 6 h, cryoprotected in 20% sucrose and embedded in the Tissue Tek OCT compound (Sakura Finetechnical, Tokyo, Japan). Coronal sections (10 µm thickness) were cut using a cryostat and were thaw-mounted onto 3-aminopropylsilane-coated glass slides. The riboprobes were synthesised by in vitro transcription from the pGEM-T Easy vector (Promega, Madison, WI, USA) containing a 690-base pair (bp) fragment of zebrafish *gpr139*-cDNA (DDBJ/EMBL/GenBank accession no, NM_001365153), representing position 1,074–1,765 bp. Sense and antisense digoxigenin (DIG)-labelled RNA probes were synthesised using MAXIscript (Ambion, Austin, TX, USA) and DIG RNA Labelling Mix (Roche Diagnostics, Mannheim, Germany). The sections were permeabilised with 0.2 M HCl for 10 min, and they were then treated with proteinase K (1 µg/ml) for 15 min, prehybridised at 58 °C for 2 h, and hybridised with DIG-labelled riboprobes (50 ng/ml) at 58 °C overnight in a humidified chamber. Following hybridisation, the sections were washed and blocked with 2% normal sheep serum. The DIG-labelled probes were detected with an alkaline phosphatase-conjugated anti-DIG antibody (Roche Diagnostics, diluted 1:500), and the chromogenic reaction was developed using 4-nitroblue tetrazolium chloride/5-bromo-4-chloro-3-indolyl-phosphate (Roche Diagnostics).

To characterise the expressing site of *gpr139* mRNA within the habenula nuclei, *gpr139* mRNA expressing cells were double-labelled with polyclonal antibodies to zebrafish Kiss1 (#PAS 15133/15134) and human GPR151 (SAB4500418, Lot#: F3111, Sigma-Aldrich), selective markers for the vHb and dHb in the zebrafish, respectively^27,33^. Specificity for the rabbit anti-zebrafish Kiss1 polyclonal antibody was previously confirmed by comparison with the expression pattern of zebrafish *kiss1* gene^27^. Specificity for the rabbit anti-human GPR151 polyclonal antibody was verified by Western blotting as well as its complete absence in GPR151-knockout mice^33^. In the zebrafish brain, the human GPR151 antibody-immunoreactive cells are seen in the dHb, and their axons projection is seen through the fasciculus retroflexus to the dorsal and ventral IPN^33^, confirming its specificity in the zebrafish. Double-fluorescent labelling of *gpr139* mRNA and Kiss1 or GPR151 immunoreactivity was performed following the procedure described previously^54^. Briefly, coronal sections of the brain (10 µm thickness) of sexually mature male zebrafish (n = 3) were hybridised with DIG-*gpr139* probes as described above. After washing and blocking of the sections with 2% normal sheep serum, the sections were incubated with a peroxidase (POD)-conjugated anti-DIG antibody (Roche Diagnostics; diluted 1:500) and DIG-labelled signals were detected using a TSA PLUS Biotin Kit (Perkin Elmer/NEN Life Science Products, Wellesley, MA) according to the manufacturer’s instructions. The sections were washed in Tris–HCl-Tween (TNT) buffer containing 0.1 M Tris–HCl pH 7.4, 0.15 M NaCl and 0.05% Triton X-100 and then incubated for 30 min in a 1:50 dilution of the Biotin Amplification Reagent (Perkin Elmer) and, DIG-labelled probes were visualised with streptavidin-conjugated Alexa Fluor 488 (1:500; Invitrogen, Eugene, OR) for 30 min. After the detection of DIG-labelled probes, the sections were treated in 10 mM citrate buffer (pH 6.0) at 62 °C for 5 min for antigen retrieval for Kiss1 or GPR151 immunohistochemistry. After the wash in PBS, the sections were incubated with Kiss1 antibody (1:500) or GPR151 antibody (1.0 µg/ml) at 4 °C for overnight followed by detection with Alexa Fluor 594-goat anti-rabbit IgG (1:400; Invitrogen, Eugene, OR).

The images of stained sections were captured and processed as described previously^54^. The sections for DIG-in situ hybridisation of *gpr139* were scanned and images were captured with a Carl Zeiss MIRAX slide scanning system (Zeiss GmbH, Göttingen, Germany) using the Mirax Viewer Image Software (3DTech, Budapest, Hungary) at a 230 nm resolution with a × 20 objective. The images of double-labelling sections were captured separately with a fluorescence microscope (ECLIPS 90i; Nikon, Tokyo, Japan) that was attached to a digital cooled CCD camera (DMX 1200, Nikon) with appropriate excitation filters for Alexa Fluor 488 (*gpr139*) and Alexa Fluor 594 (Kiss1 and GPR151), and computer software (NIS Elements D3.2; Nikon) was used to superimpose the two images. The red channel was then converted to magenta, and brightness and contrast adjustments were made in Adobe Photoshop CC (Adobe, San Jose, CA, USA).

### Dual-luciferase reporter assay

JNJ-63533054 (also known as compound 7c), (S)-3-chloro-N-(2-oxo-2-((1-phenylethyl)amino ethyl) benzamide (Axon Medchem, Groningen, Netherlands) is a commercially available agonist for human and other mammalian GPR139 (Dvorak et al. 2015; Liu et al. 2015). To confirm if JNJ-63533054 acts as an agonist to zebrafish GPR139, the binding of JNJ-63533054 to the zebrafish GPR139 was examined by a dual-luciferase reporter gene assay. The binding affinity of JNJ-63533054 for human GPR139 was also tested as an assay control.

A pcDNA3.1( +) expression vector containing an open reading frame of zebrafish *gpr139* cDNA (pc_zfGPR139, Clone ID, ODa58136D) and human *GPR139* (pc_hGPR139, GenBank accession number NM_001002911.3; Clone ID, OHu30000) were obtained from GenScript Ltd (Hong Kong). HEK293-T cells were maintained in Dulbecco’s modified Eagle’s medium (DMEM; GIBCO, Auckland, NZ) supplemented with 10% fetal bovine serum (FBS), 0.1 × penicillin–streptomycin solution (iDNA, Kuala Lumpur, Malaysia) under 5% CO_2_. One day before transfection, cells were plated in 24-well plates in the media without penicillin–streptomycin. Co-transfection of pc_zfGPR139 or pc_hGPR139 (100 ng/well) with pSRE-Luc (100 ng/well; Stratagene, La Jolla, CA), and pRL-TK vectors (25 ng/well; Promega, Madison, WI) was carried out with Lipofectamine 2000 transfection reagent (Invitrogen; Thermo Fisher Scientific, Inc.) overnight according to the manufacturer’s instructions. The cells were serum starved in the media with 0.5% FBS for 18–20 h, and then treated with the vehicle (control) or various concentrations (4 ~ 128 nM in 0.5% DMSO) of JNJ-63533054 in the media for 6 h. The cells were harvested and lysed with passive lysis buffer, then analysed immediately using a 96-well plate luminometer (Infinite M200pro, Tecan, Switzerland). Luciferase activity and *Renilla* luciferase in the cell extracts was determined using Dual–Luciferase Reporter Assay System (Promega) according to the manufacturer’s instruction. The value of luciferase for each lysate was normalised to the *Renilla* luciferase activity. The relative transcriptional activity was converted to fold induction above the corresponding vehicle control value (n-fold). A nonlinear regression was used to determine the agonist EC50 values (concentration of the agonist that produced the half-maximal response).

### Intraperitoneal administration of GPR139 selective agonist, JNJ-63533054

JNJ-63533054 is a brain penetrant GPR139 selective agonist that crosses the blood–brain barrier in rats^1,9^. Hence, it was delivered via intraperitoneal administration in this study. The doses for JNJ-63533054 were chosen based on previous in vivo and in vitro assays conducted in rats^1^. As JNJ-63533054 is a hydrophobic compound (Dvorak et al., 2015; Liu et al., 2015), it was solubilised in dimethyl sulfoxide (DMSO). Therefore, the final concentrations of DMSO containing in JNJ-63533054 solution were minimised (0.01%, 0.1% and 1% for 0.1, 1 and 10 µg/g body weight, BW, respectively in locomotor activity and fear response experiment and 0.2% and 2% for 0.1 and 1 µg/g BW, respectively in fear memory consolidation experiment), which have previously been validated as the range of doses that cause no significant variation in behaviours in larval and adult zebrafish^55^. Intraperitoneal administration was carried out according to the protocol previously reported^56^. Briefly, fish were anesthetised with benzocaine (0.1 g benzocaine/200 mL water) and placed on a sponge soaked with water. The fish were intraperitoneally administered with 5 µL of either JNJ-63533054 dissolved in DMSO solution (0.01%, 0.1%. and 1% in fear response experiment and 0.2 and 2% in fear memory consolidation experiment) or water containing the same concentrations of DMSO into the peritoneal cavity by a 35G NanoFil Bevelled Needle attached with 10 μl NanoFil microsyringe (World Precision Instruments, Sarasota, FL). After administration, the fish were individually transferred to a tank for recovery from the anesthetisation.

### Effect of JNJ-63533054 on locomotor activity

The effect of JNJ-63533054 on locomotor activity was examined. Fish (n = 5) were administered with JNJ-63533054 as described above. After recovery from the anesthetisation (~ 5 min), the fish was transferred to the observation tank [361 mm length (L), 218 mm width (W), 256 mm height (H)] and the locomotion was recorded for 6 min. Side view of fish behaviour was recorded by a video camera (Handycam DCR-SX83E, Sony), between 1100 and 1600 h with a similar temperature (28 ± 0.5 °C) and lighting (802.4 lx illumination) condition to the home tank. Captured video data were analysed using automated tracking software, LoliTrack 2.0 (Loligo Systems, Denmark).

### Alarm substance (AS)-induced fear response

AS is a potential stimulus for avoidance conditioning that sensitises anxiety-like behaviour after a single exposure and elicits behaviours, such as erratic movements and freezing. The effect of JNJ-63533054 on aversive response was examined using the AS-induced fear response model^57^. AS solution was prepared according to the protocol implemented previously ^27^. Briefly, male fish were anesthetised by submerging them in ice-cold water, and fifteen shallow cuts were made on the right trunk of the zebrafish with a razor blade, and the cuts were washed with 5 mL ice-cold DW. This was then repeated on the left trunk of the fish to obtain a total of 10 mL AS solution per fish.

Fish (n = 10 per group) were intraperitoneally administered with three doses (0.1, 1, and 10 µg/g BW) of JNJ-63533054 or DW using a microinjector as described above, and individually transferred to a tank (361 mm L, 218 mm W), 256 mm H) for recovery from the anesthetisation. After the recovery (~ 10 min), the general locomotion (including distance travel and latency) of the fish were recorded for 10-min from the side-view. After 20 min of further acclimatisation, 1 mL of AS or DW was applied into the tank through a glass capillary positioned at the corner of the tank about 0.5 cm below the water level. The mean time for introduction through the capillary was 5 s. Immediately, after the exposure to AS/DW, following reference parameters of swimming behaviours of the fish (n = 10 per group) including the total distance swam, total freezing time (total absence of movement for 1 s or longer), total time spent, and the total swimming velocity that was only measured at the bottom of a tank as the fear responses occur mainly on the bottom of the tank upon AS delivery^57^.

### Effect of JNJ-63533054 on fear memory consolidation

**Figure 7:**
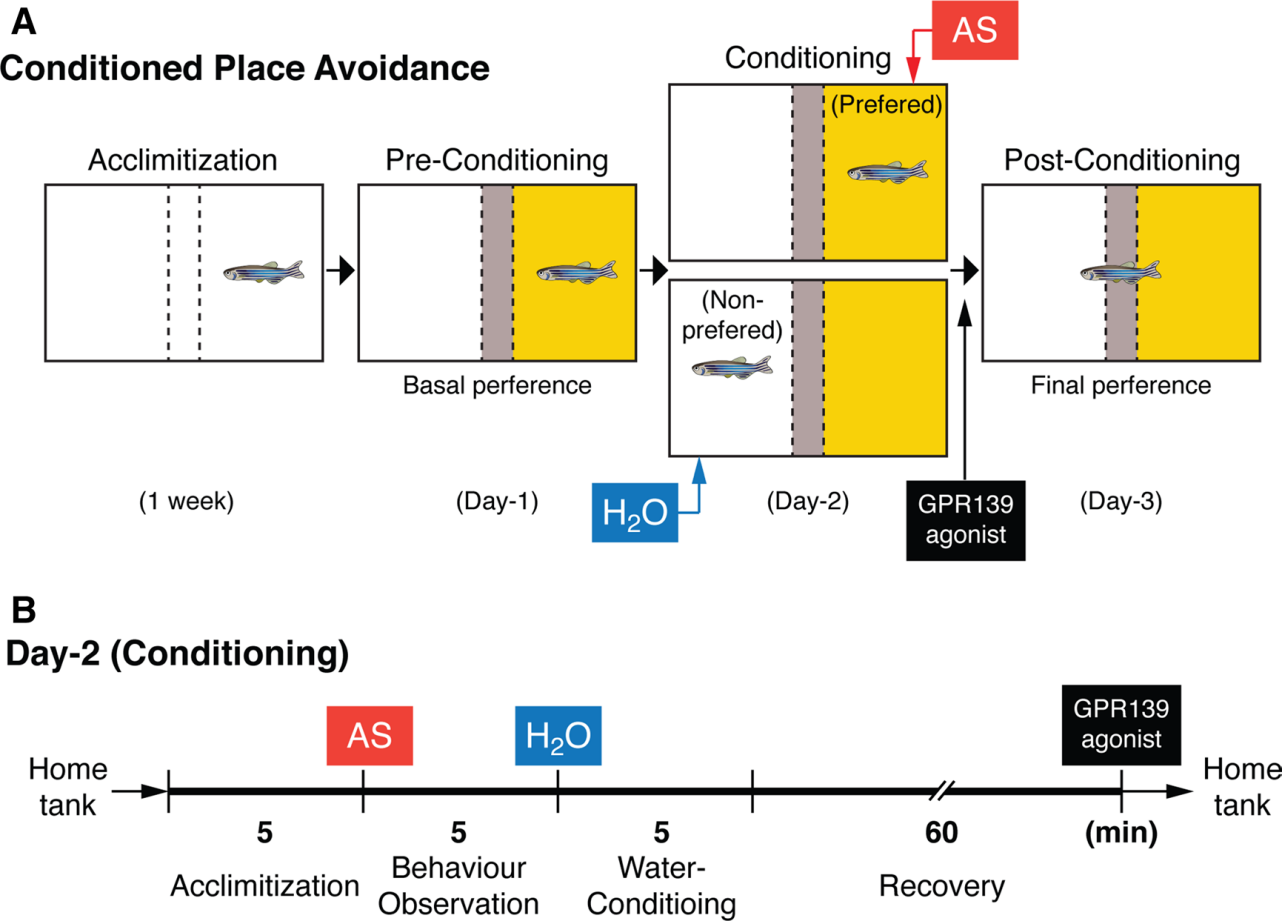
Alarm substance-induced fear conditioning and GPR139 agonist treatment timeline during the conditioning. (**A**) Schematic drawing of conspecific alarm substance (AS)-induced fear conditioning paradigm. Adopted by Sivalingam et al. (2020)^30^. During pre-conditioning (Day-1), fish was given a choice for their preferred colour, either yellow or white coloured compartment (basal preference). After conditioning to AS-induced fear responses (Day-2), their change in preference was assessed based on their total time spent in AS-paired (originally preferred) compartment as compared to the initial preference (Day-3). (**B**) On Day-2 (conditioning phase), fish were individually placed into the compartment, and after 5 min of acclimatisation time, AS was delivered in water followed by 5-min of video recording. The fish was then immediately transferred into the non-preferred compartment of the new experimental tank and exposed with 2 mL of distilled water (H_2_O) for 5-min. In order to avoid the conditioning to AS-induced fear but not to intraperitoneal-administration and handling stress, GPR139 agonist was then intraperitoneally injected after 60 min of the recovery from the conditioned stimuli (in total 65-min from the fear conditioning), and fish were transferred to their respective home tank.

Effect of JNJ-63533054 on fear memory consolidation was assessed using an AS-induced conditioned place avoidance paradigm that was previously established by Maximino and co-workers^32^ with some modifications as described below (Fig. 7).

#### AS-paired conditioned place avoidance paradigm

##### Experimental apparatus

A test tank (31 cm L × 16 cm W × 20 cm H) divided into three-chambers using a lightweight board made of a corrugated plastic with yellow or white (13 cm L × 16 cm W × 20 cm H) and a grey central partition (31 cm L × 5 cm W × 20 cm H) and two sliding guillotine-type doors (16 cm × 20 cm) (Fig. 7A). The partition allows the water to move throughout the tank and the AS solution can be diffused throughout the tank in a minutes after application^58^. Another tank (31 cm L × 16 cm W × 20 cm H) used as an acclimatising tank, was similarly divided as above but by a transparent divider to allow conspecific to visualise in order to minimise isolation stress. The top view of fish behaviour was recorded by a video camera (positioned approximately 1 m above the tank), between 1100 and 1600 h with the similar temperature (28 ± 0.5 °C) and lighting (802.4 lx illumination) condition to the home tank. Captured video data were analysed using automated tracking software, LoliTrack 2.0 (Loligo Systems).

##### Acclimatisation

A week prior to the behavioural study, fish were randomly taken from the home tank and transferred to an acclimatising tank. To reduce the potential handling stress, 5-min of net handling was applied to fish once daily throughout the acclimatising period. The fish were habituated to netting and transferring, in which the fish was netted and transferred to a beaker during changing of water every day throughout the acclimatisation period. However, the condition of water, temperature and light were maintained the same as in the home tanks.

##### Pre-conditioning phase

On the day of conditioning, the fish was individually placed into the central compartment (grey) of the apparatus. After 30s of familiarisation period, the separators which block the yellow and white compartment respectively were removed to enable the fish to move freely for 5 min followed by 6 min of video recording to assess a basal preference of the fish by measuring the time spent in each compartment.

##### Conditioning

During the conditioning phase, fish were individually placed into the compartment that was initially chosen as the preferred compartment (> 50% time spent) during the pre-conditioning phase, and after 5 min of settling time, AS was delivered in water followed by 5-min of video recording (Fig. 7B). Since the chemical nature of AS has not been fully characterised^59,60^, the exact concentration of AS could not be determined. However, the ratio (2 ml of AS in 5 L of water) applied consistently induced typical fear-like responses throughout the experiments. The fear parameters including erratic movement and freezing time were assessed as described above. The fish was then immediately transferred into the non-preferred compartment of the new experimental tank and 2 mL of DW was added into the tank. In order to avoid the conditioning to AS-induced fear but not to intraperitoneal-administration and handling stress, GPR139 agonist was then intraperitoneally injected after 60 min of the recovery from the AS-induced stimuli (in total 65-min after the fear conditioning), and fish were transferred to their respective home tank.

##### Post-conditioning

On the 3rd day of the test, after the conditioning period, each fish was placed in the centre compartment (grey) before the separator was removed. During the post-conditioning period, the avoidance of the AS-conditioned compartment was assessed by comparing the time spent in the AS-conditioned and unconditioned compartments for 6 min. In addition, fear-related responses of the fish in the AS-conditioned compartment were also assessed as described above.

### Statistics

All behavioural data were analysed using the Statistical Package for the Social Sciences (SPSS, Version 24, IBM). All behavioural endpoints data were expressed as means ± standard error of the mean (S.E.M.). For the dual-reporter luciferase assay, the results were analysed using Prism 9.0.0 (GraphPad Software, Inc., San Diego, USA) and representatives of three independent experiments in duplicate. Luciferase responses were normalised as indicated and the concentration–response curves were fitted using nonlinear regression in a sigmoidal model with variable slope according to the standard procedure provided by Graph Pad. All graphs are made in Prism 9.0.0. The dose-dependent effect of JNJ63533054 on fear response was assessed using one-way analysis of variance (ANOVA) followed by.

Tukey’s multiple comparison test with a single pooled variance and effect sizes were reported as generalised eta squared (gεs) with 95% confidence interval level. Basal preference of fish and statistical significance between controls and experimental groups for assessing post-conditioning period (total time spent and total freezing time) was assessed by independent samples *t*-test, and effect sizes were reported as Cohen’s *d*. We also conducted the Mann–Whitney U test to analyse the total time spent in the centre compartment. *P* < 0.05 is considered statistically significant.

## Supplementary Information

Supplementary Legends.

Supplementary Figures.
