# Supplementary material for: Habenula GPR139 is associated with fear learning in the zebrafish
Source: Sci Rep. 2021 Mar 10;11:5549. doi: 10.1038/s41598-021-85002-1 (PMC7946892; doi:10.1038/s41598-021-85002-1)
Supplement: Supplementary file 1 — Supplementary Legends. [file 41598_2021_85002_MOESM1_ESM.docx]

**Supplementary Figs. S1 and S2. *In situ* hybridisation localisation of *gpr139* mRNA in the brain of zebrafish.** In the top panel, lines on a schematic sagittal drawing view of the zebrafish brain indicate levels of coronal sections (a–i, sense probe; a’–i’, antisense probe). Abbreviations: OB, olfactory bulb; TEL, telencephalon; POA, preoptic area; OC, optic chiasma; HYP, hypothalamus; HB, habenula; OT, optic tectum; MB, midbrain; CEL, cerebellum; MO, medulla oblongata. Scale bars, Fig. S1, 100 μm; Fig. S2, 200 μm,
