# Supplementary material for: Habenula GPR139 is associated with fear learning in the zebrafish
Source: Sci Rep. 2021 Mar 10;11:5549. doi: 10.1038/s41598-021-85002-1 (PMC7946892; doi:10.1038/s41598-021-85002-1)

Supplementary Information

Habenula GPR139 is associated with fear learning in the zebrafish

Nisa Roy, Satoshi Ogawa, Roshan Maniam, and Ishwar Parhar*

Supplementary Fig. S1

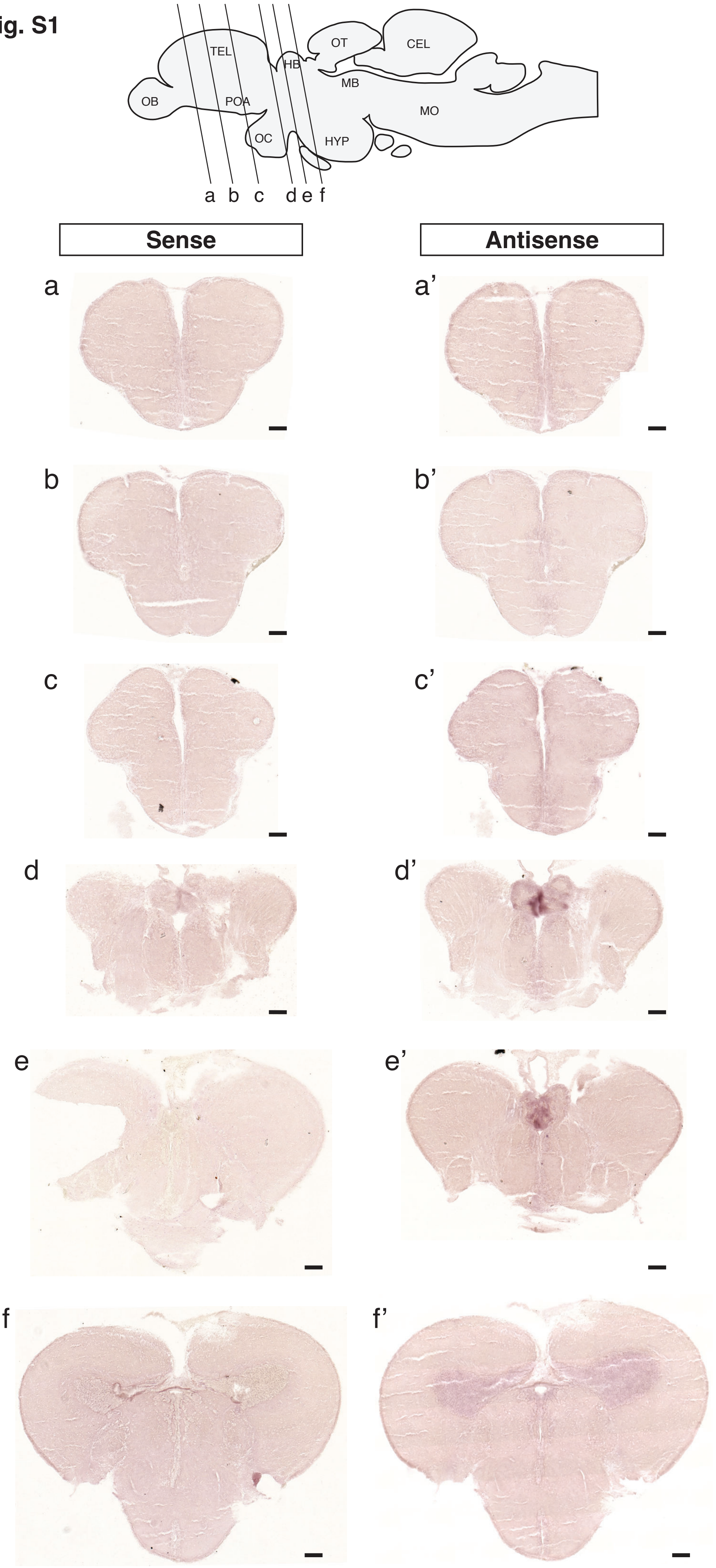

Supplementary Fig. S2

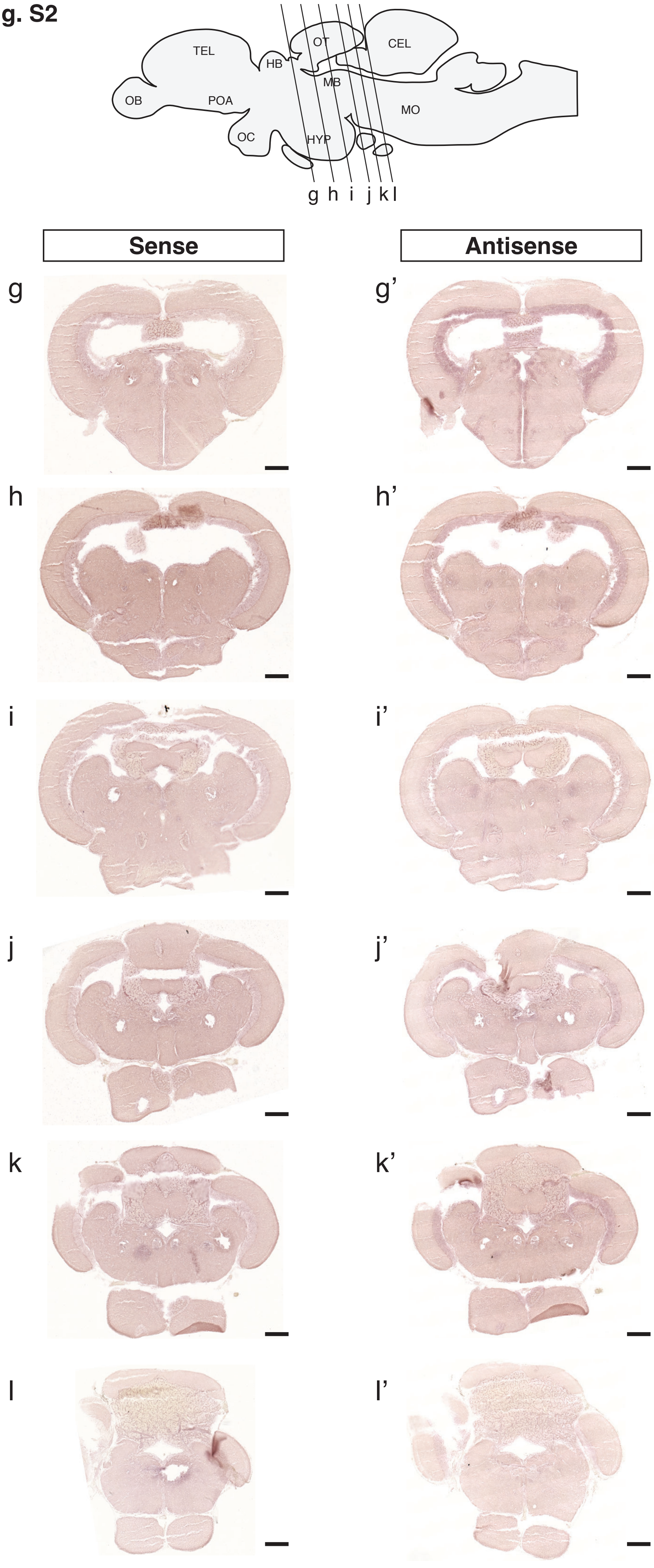

Supplement: Supplementary file 2 — Supplementary Figures. [file 41598_2021_85002_MOESM2_ESM.pdf]
